# Mutations in sphingolipid metabolism genes are associated with ADHD

**DOI:** 10.1038/s41398-020-00881-8

**Published:** 2020-07-13

**Authors:** Marcela Henriquez-Henriquez, Maria T. Acosta, Ariel F. Martinez, Jorge I. Vélez, Francisco Lopera, David Pineda, Juan D. Palacio, Teresa Quiroga, Tilla S. Worgall, Richard J. Deckelbaum, Claudio Mastronardi, Brooke S. G. Molina, the MTA Cooperative Group, Benedetto Vitiello, Joanne B. Severe, Peter S. Jensen, L. Eugene Arnold, Kimberly Hoagwood, John Richters, Donald R. Vereen, Stephen P. Hinshaw, Glen R. Elliott, Karen C. Wells, Jeffery N. Epstein, Desiree W. Murray, C. Keith Conners, John March, James Swanson, Timothy Wigal, Dennis P. Cantwell, Howard B. Abikoff, Lily Hechtman, Laurence L. Greenhill, Jeffrey H. Newcorn, Brooke S. G. Molina, Betsy Hoza, William E. Pelham, Robert D. Gibbons, Sue Marcus, Kwan Hur, Helena C. Kraemer, Thomas Hanley, Karen Stern, Mauricio Arcos-Burgos, Maximilian Muenke

**Affiliations:** 1 Department of Clinical Laboratories, School of Medicine, Pontificia Universidad Católica de Chile, Santiago, Chile; 2 ELSA Clinical Laboratories (IntegraMedica, part of Bupa), Santiago de Chile, Chile; 3 Medical Genetics Branch, National Human Genome Research Institute, National Institutes of Health, Bethesda, MD USA; 4 Universidad del Norte, Barranquilla, Colombia; 5 Neuroscience Research Group, University of Antioquia, Medellin, Colombia; 6 Department of Pathology and Cell Biology, Columbia University, New York, NY USA; 7 Department of Pediatrics, Institute of Human Nutrition, College of Physicians and Surgeons, Columbia University, New York, NY USA; 8 Neuroscience Group (NeurUROS), Institute of Translational Medicine, School of Medicine and Health Sciences, School of Medicine and Health Sciences, Universidad del Rosario, Bogotá, Colombia; 9 Departments of Psychiatry, Psychology, and Pediatrics, University of Pittsburgh, Pittsburgh, PA USA; 10 Grupo de Investigación en Psiquiatría (GIPSI), Departamento de Psiquiatría, Instituto de Investigaciones Me´dicas, Facultad de Medicina, Universidad de Antioquia, Medelli´n, Colombia; 11 Child & Adolescent Treatment and Preventive Interventions Research Branch, National Institute of Mental Health, National Institutes of Health, Bethesda, MD USA; 12 Division of Services and Intervention Research, National Institute of Mental Health, National Institutes of Health, Bethesda, MD USA; 13 The REACH Institute, New York, NY USA; 14 Department of Psychiatry and Psychology, Mayo Clinic, Rochester, MN USA; 15 Center for Psychiatry and Behavioral Health, The Ohio State University, Columbus, OH USA; 16 Department of Child Psychiatry, Columbia University, New York, NY USA; 17 National Institute of Nursing Research, National Institutes of Health, Bethesda, MD USA; 18 National Institute of Drug Abuse, National Institutes of Health, Bethesda, MD USA; 19 The Institute of Human Development, University of California Berkeley, Berkeley, CA USA; 20 Department of Psychiatry, University of California San Francisco, San Francisco, CA USA; 21 Children’s Health Council, Palo Alto, CA USA; 22 Department of Psychiatry and Behavioral Sciences, Duke University School of Medicine, Durham, NC USA; 23 Department of Pediatrics, Cincinnati Children’s Hospital Medical Center, Cincinnati, OH USA; 24 The Sanford School of Public Policy, Duke University, Durham, NC USA; 25 Frank Porter Graham Child Development Institute, University of North Carolina at Chapel Hill, Chapel Hill, NC USA; 26 Department of Pediatrics, University of California, Irvine School of Medicine, Irvine, CA USA; 27 Avida Inc., Newport Beach, CA USA; 28 Department of Psychiatry and Behavioral Sciences, Neuropsychiatric Institute, University of California, Los Angeles School of Medicine, Los Angeles, CA USA; 29 Department of Child and Adolescent Psychiatry, New York University Grossman School of Medicine, New York, NY USA; 30 Division of Child Psychiatry, Montreal Children’s Hospital, McGill University, Montreal, QC Canada; 31 Division of Child and Adolescent Psychiatry, Department of Psychiatry, Columbia University, New York, NY USA; 32 Department of Psychiatry, Icahn School of Medicine at Mount Sinai, New York, NY USA; 33 Department of Psychological Science, University of Vermont, Burlington, VT USA; 34 Department of Psychology, Florida International University, Miami, FL USA; 35 Departments of Medicine, Public Health Sciences and Comparative Human Development, The University of Chicago, Chicago, IL USA; 36 Icahn School of Medicine at Mount Sinai, New York, NY USA; 37 Department of Psychiatry, University of Illinois at Chicago, Chicago, IL USA; 38 Department: Psychiatry and Behavioral Sciences, Stanford University, Stanford, CA USA; 39 Office of Special Education Programs, US Department of Education, Washington, DC USA; 40 Office of Juvenile Justice and Delinquency Prevention, US Department of Justice, Washington, DC USA

**Keywords:** ADHD, Predictive markers, Clinical genetics

## Abstract

Attention deficit hyperactivity disorder (ADHD) is the most prevalent neurodevelopmental disorder in children, with genetic factors accounting for 75–80% of the phenotypic variance. Recent studies have suggested that ADHD patients might present with atypical central myelination that can persist into adulthood. Given the essential role of sphingolipids in myelin formation and maintenance, we explored genetic variation in sphingolipid metabolism genes for association with ADHD risk. Whole-exome genotyping was performed in three independent cohorts from disparate regions of the world, for a total of 1520 genotyped subjects. Cohort 1 (MTA (Multimodal Treatment study of children with ADHD) sample, 371 subjects) was analyzed as the discovery cohort, while cohorts 2 (Paisa sample, 298 subjects) and 3 (US sample, 851 subjects) were used for replication. A set of 58 genes was manually curated based on their roles in sphingolipid metabolism. A targeted exploration for association between ADHD and 137 markers encoding for common and rare potentially functional allelic variants in this set of genes was performed in the screening cohort. Single- and multi-locus additive, dominant and recessive linear mixed-effect models were used. During discovery, we found statistically significant associations between ADHD and variants in eight genes (*GALC*, *CERS6*, *SMPD1*, *SMPDL3B*, *CERS2*, *FADS3*, *ELOVL5*, and *CERK*). Successful local replication for associations with variants in *GALC*, *SMPD1*, and *CERS6* was demonstrated in both replication cohorts. Variants rs35785620, rs143078230, rs398607, and rs1805078, associated with ADHD in the discovery or replication cohorts, correspond to missense mutations with predicted deleterious effects. Expression quantitative trait loci analysis revealed an association between rs398607 and increased *GALC* expression in the cerebellum.

## Introduction

Attention deficit hyperactivity disorder (ADHD) is defined as a neurodevelopmental condition characterized by persistent, cross-situational and developmentally inappropriate levels of inattention, hyperactivity, and impulsiveness that leads to various degrees of functional impairment^1^. It is the most common neuro-behavioral disorder in childhood, affecting 5.29–7.1% of children and adolescents^2^. Prevalence in adults is also high, with best estimates between 2.5 and 2.8% worldwide^3,4^.

Genetic factors account for ~75–80% of the phenotypic variance of the ADHD phenotype^5–7^. Interesting results have emerged from studies of candidate genes involved in the monoamine neurotransmitter systems, which had been implicated in the pathophysiology of ADHD by the mechanisms of action of drugs used in clinical management. Family-based and case–control studies of candidate genes have replicated significant linkage and/or association between ADHD and variants in dopamine receptors (*DRD4*, *DRD5*), dopamine transporter (*SLC6A3*), serotonin transporter (*SLC6A4*), serotonin receptor (*HTR1B*), and proteins involved in synaptic transmission (*SNAP25*, *LPHN3*)^6,8–16^, all of them contributing to small- to medium-sized effects. The first 12 genome-wide significant ADHD risk loci were published recently^17^. Several of the identified loci are located in or near genes (e.g., *FOXP2*, *SORCS3*, and *DUSP6*) that implicate neurodevelopmental processes likely to be relevant to ADHD pathogenesis. Historically, the lack of success in identifying genome-wide significant variants supports the complex multifactorial etiology of ADHD and likely reflects important biases in patient ascertainment and phenotyping strategies. Therefore, continued efforts are required to elucidate the missing heritability of ADHD.

Neuroimaging studies have suggested white/gray matter anomalies in the prefrontal cortex, temporo-parietal regions, the striatum, and the cerebellum in ADHD patients^18–20^. Longitudinal neuroimaging studies targeting white and gray matter alterations have led to the proposition that ADHD involves a lag in brain maturation that eventually normalizes^21^. Additional evidence suggests that atypical myelination and gray matter anomalies might persist into adulthood in patients with ADHD^22,23^. Based on this evidence, myelination and neurogenesis appears to be highly attractive novel targets for genomic/metabolomic studies in ADHD.

Sphingolipids encompass a complex range of membrane lipids in which a fatty acid is linked to a sphingosine carbon backbone. Depending on the sphingosine head group, they can be further classified into ceramides (no head group), phosphosphingolipids (mostly sphingomyelins), or glycosphingolipids (cerebrosides and the more complex gangliosides)^24^. Sphingolipids are important structural and signaling molecules that affect processes such as neuronal and glial proliferation, differentiation and apoptosis, nerve impulse generation and propagation, and neurotransmitter release^25–27^. Cell and animal models underscore the key function of sphingolipids in neurite growth and myelination in the central nervous system (CNS)^28–30^. Deficiency of ceramide synthase-2, an enzyme that catalyzes the synthesis of sphingolipids with very long acyl chains (C20–C26), results in 50% loss of compacted myelin and 80% loss of CNS myelin basic protein^30^. Similarly, mice with ceramide synthase-1 deficiency (enzyme specific for C18:0 acyl chains) show a 60% reduction in the levels of neuronal gangliosides and oligodendrocytic myelin-associated glycoprotein in the cerebellum and forebrain^28^.

The role of sphingolipids in ADHD pathogenesis has not been explored. Recently, a pilot study characterizing the serum sphingolipid profiles of ADHD patients revealed decreased levels of sphingomyelins and specific long-chain ceramides. These preliminary results also suggested that sphingolipids might eventually become an endophenotype for ADHD^31^, opening the field to the search of new genetic risk variants in genes participating in sphingolipid metabolism. Here, we investigated 1520 genotyped individuals from three independent and geographically disparate populations to target potentially functional variants in 58 genes participating in sphingolipid metabolism. Our results provide the first evidence of a link between sphingolipid metabolism and ADHD susceptibility.

## Patients and methods

### Patients

#### MTA sample

The Multimodal Treatment study of children with ADHD (MTA) was designed to evaluate long-term effects of treatments for ADHD in a 14-month randomized controlled trial of 579 children meeting the Diagnostic and Statistical Manual of Mental Disorders, Fourth Edition (DSM-IV)^32^ criteria for ADHD using the Diagnostic Interview Schedule for Children-Parent Version (DISC-P), supplemented with teacher report of symptoms. The DISC-P was administered at entry (in childhood) and at each of the prospective follow-up assessments, including the 6-year follow-up when the participants were between 13.0 and 15.9 years of age. After the initial 14-month treatment-by-protocol phase, the study continued as an observational follow-up into early adulthood, in which self-selected use of treatments and other variables were monitored^33^. The clinical and demographical characteristics of the sample, along with the recruitment procedures, have been extensively described^34^. Mean age of children at recruitment was 8.5 years (range 7–10 years) and 80.3% were males (*n* = 465). Ethnic composition of the sample included 61.5% Caucasian, 17.5% African American, 10.6% Hispanic, 1.5% Asian, and 8.9% of other race and ethnic minorities. Exclusion criteria for the MTA cohort are reported in the original study^34^. These criteria were limited to situations that would prevent families’ full participation in assessments or treatment, or that might require additional treatments incompatible with study treatments. The presence of comorbid conditions, such as oppositional defiant disorder (ODD), conduct disorder (CD), internalizing disorders, or specific learning disabilities, did not lead to exclusions per se as an important aim of previous studies was to examine their interactions with treatment outcomes^34–37^.

A local normative comparison group (LNCG) of 289 randomly selected classmates matched for grade and sex was added when participants were between 9 and 12 years old. Participants were diagnosed in childhood using the Diagnostic Interview Schedule for Children-Parent Version (DISC-P), which was administered at entry and at the prospective follow-up assessments. Outcomes in childhood (14, 24, and 36 months after baseline), adolescence (6 and 8 years after baseline), and adulthood (up to 16 years after baseline) have been reported^34,36–38^. For the current study, only 371 subjects (280 males and 91 females) were available for genotyping, consisting of 232/579 from the MTA group and 139/289 from the LNCG group.

The MTA study is a cooperative effort of six independent research teams in collaboration with the Division of Services and Intervention Research, National Institute of Mental Health, and the Office of Special Education Programs, US Department of Education, Washington, DC. Research was conducted in accordance with the ethical guidelines of local Institutional Review Boards (IRBs) and the National Institutes of Health’s Office for Protection from Research Risks, Bethesda, MD. Patients were recruited under clinicaltrials.gov registration NCT00000388.

### Replication

#### Paisa cohort

This cohort consists of 1176 persons (adults and children) from 18 extended multigenerational and 136 nuclear Paisa families inhabiting the Medellin metropolitan area in the State of Antioquia, Colombia (mean age 28 ± 17 years, 45% males). The detailed clinical and demographic description of the sample and the recruitment procedures have been published elsewhere^39^. Parents underwent a full psychiatric structured interview regarding their offspring (DICA-IV-P, Spanish version translated with permission from W. Reich)^40^. In addition, adult participants were assessed using the Composite International Diagnostic Interview as well as the Disruptive Behavior Disorders module from the DICA-IV-P modified for retrospective use^40^. ADHD status was defined by the best estimate method (consensus diagnosis, evaluating all available clinical information). ADHD in these extended Paisa families is highly comorbid with CD, ODD, and nicotine and alcohol abuse^39^. The comorbidity pattern and the large dense pedigrees of the sample have been particularly useful to identify genes conferring susceptibility to ADHD in previous molecular genetic studies^12,13,39,41–43^. Studies in the Paisa cohort were approved by the Ethics Committee of the University of Antioquia (Medellin, Colombia) and the National Human Genome Research Institute’s IRB office (Bethesda, MD), and informed consent was obtained from all subjects in accordance with the Declaration of Helsinki. Patients were recruited under NHGRI protocol 00-HG-0058 (clinicaltrials.gov: NCT00046059).

#### US cohort

Clinical and demographic characteristics of this sample, along with a detailed description of the recruitment protocol, have been published elsewhere^44^. Briefly, participants were recruited by advertising in national ADHD-related publications in the USA and on the NIH/NHGRI web page (https://www.genome.gov). Eligible families included a proband with a diagnosis of ADHD who was between 7 and 18 years of age at enrollment with at least one sibling (either affected or not). Additionally, at least one parent had to be available to participate with information accessible regarding both parents. Interested families underwent an exhaustive screening evaluation comprising questions regarding pregnancy and birth history for the proband and siblings and rating scales: the Vanderbilt Assessment Scale for Parents^45^, used for all family members; the Wender Utah Rating Scale^46^ and Conners Adult ADHD Rating Scale^47^, applied exclusively in adults; the Strengths and Weaknesses of Attention and Normal Behavior^48^, for children and adolescents. Additionally, parents underwent a full structured psychiatric interview regarding each offspring (DICA-IV-P)^40^ and all siblings 18 years or older responded to the Structured Clinical Interview for DSM-IV^49^. Questionnaires and eligibility criteria were reviewed by a clinical team consisting of a registered nurse coordinator, two registered nurses, and a clinical social worker, all with extensive training in behavioral conditions and ADHD research. Pedigrees were obtained from all families. Exclusion criteria included the following: (i) Bilineal families (both parents affected with ADHD), (ii) families with probands that met the DSM-IV^32^ criteria for Tourette’s disorder, obsessive compulsive disorder, pervasive developmental disorders, psychotic disorders, mood disorders with psychotic features, post-traumatic stress disorder, or (iii) prior diagnosis of lead toxicity, neurological conditions, known genetic syndromes, mental retardation, hydrocephaly, known prenatal drug exposure, cardiac surgery, or prematurity (birth weight below 2500 g). The total sample consisted of 1010 individuals (49.6% affected by ADHD, 55% males, 37.2% of them under 17 years at the enrollment). The study and consent forms were reviewed and approved by the National Human Genome Research Institute’s IRB office. Patients were recruited under NHGRI protocol 00-HG-0058 (clinicaltrials.gov: NCT00046059).

### Whole-exome genotyping

DNA for genotyping was extracted from whole blood. Whole-exome genotyping was performed in 371 subjects (280 males and 91 females) from the MTA cohort (232/579 subjects from MTA and 139/289 subjects from LNCG). From the Paisa cohort, whole-exome genotyping comprised 298 participants, consisting of 159/1176 (13.5%) ADHD affected and 139/1176 (11.8%) unaffected controls. From the US cohort, whole-genome genotyping comprised 851 individuals. Genomic DNA was whole-exome genotyped using the Illumina® HumanExome BeadChip-12v1_A. This single-nucleotide polymorphism (SNP) chip covers >240,000 putatively functional exonic variants from over 12,000 individuals representing diverse populations (including European, African, Chinese, and Hispanic individuals) and a range of common conditions, such as type 2 diabetes, cancer, metabolic, and psychiatric disorders. In addition to coding variation, the HumanExome BeadChip-12v1_A chip covers SNPs in canonical splice sites (10,675) and promoter regions (7012). No 3′-untranslated region variants are represented. To test genotyping reliability and quality, one individual sample was duplicated. Processed and raw intensity signals for the array data can be accessed at GEO (https://www.ncbi.nlm.nih.gov/geo, accession no. GSE112652).

### Genetic, statistical, and bioinformatics analyses

#### Quality control, filtering, and classification of coding variants

Genetic data were imported to Golden Helix^®^’s SVS 8.3.0, and quality control was performed using the following criteria: (i) fitting to Hardy–Weinberg equilibrium with *P* values >0.05/*m* (where *m* is the number of markers included for analysis); (ii) a minimum genotype call rate of 90%, that is, at least 90% of individuals in the sample have available genotypes; (iii) and presence of two alleles. Markers not meeting any of these criteria were excluded from analyses. Genotype and allelic frequencies were estimated by maximum likelihood. Variants with a minor allele frequency (MAF) ≥ 0.01 were classified as common and rare otherwise, according to previous recommendations^50–52^. Exonic variants with potential functional effect were identified using the annotations in the database for nonsynonymous SNPs’ functional predictions (dbNSFP, GRCh37/hg19 genome assembly)^53^. This filter uses SIFT, Provean, PolyPhen-2, Mutation Taster, Mutation Assessor, Gerp^++^, and PhyloP to predict a variant’s deleterious effect^54–58^ and is fully implemented in the SVS 8.3.0 Variant Classification module.

#### Gene selection for targeted analysis

A set of 58 genes was manually curated based on their roles in sphingolipid metabolism (Table 1). The selected genes encode for enzymes involved in the de novo synthesis or recycling of sphingolipids. A subset of genes involved in fatty acid elongation/desaturation was also included because of the direct interplay between sphingolipid and fatty acid metabolic pathways^59–61^. Associations/trends between ADHD and regions containing some of the genes included in the set have been observed in previous genome-wide association study (GWAS)/copy number variation (CNV) studies (see Table 1)^15,62–69^. With the exception of *FADS1* and *FADS2* (which encode fatty acid desaturases 1 and 2, respectively), no other candidate–gene studies have explored possible associations between ADHD risk and the remaining 56 genes examined in this study.

**Table 1:** Set of 58 genes selected for targeted analysis.

Enzyme	Gene	Previous association with ADHD (ref.)
Serine-palmitoyl transferase	*SPTLC1*	Association/trend with gene-related region^62,67^
	*SPTLC2*	
	*SPTLC3*	Association/trend with gene-related region^62,67^
	*SPTSSA*	
	*SPTSSB*	Association with gene-related CNV^69^
3-Ketodihydrosphingosine reductase	*KDSR*	
Ceramide synthase	*CERS1*	
	*CERS2*	
	*CERS3*	
	*CERS4*	
	*CERS5*	
	*CERS6*	
Dihydroceramide desaturase	*DEGS1*	
	*DEGS2*	
Fatty acid elongases	*ELOVL1*	
	*ELOVL2*	
	*ELOVL3*	
	*ELOVL4*	
	*ELOVL5*	
	*ELOVL6*	Trend associated SNP^66^
	*ELOVL7*	Gene-related CNV^65^
Ceramide kinase	*CERK*	
Sphingomyelin synthase	SMS1	
	SMS2	
Sphingomyelinase	*SMPD1*	Gene-related CNV^69^
	*SMPD2*	
	*SMPD3*	
	*SMPD4*	Association with gene-related region^68^
	*SMPDL3A*	Association with gene-related region^67^
	*SMPDL3B*	
	*ENPP7*	
UDP-glucose ceramide glucosyltransferase	*UGCG*	Association with gene-related region^67^
UDP-galactosyltransferase 8	*UGT8*	
Galactosylceramidase	*GALC*	
Beta-1-4-galactosyltransferase 6	*B4GALT6*	Association with gene-related region^67^
Galactose-3-O-sulfotransferase	*GAL3ST1*	
Alkaline ceramidase	*ACER1*	
	*ACER2*	
	*ACER3*	Association/trend with gene-related region^12,64^
Acid ceramidase	*ASAH1*	
	*ASAH2*	
Sphingosine kinase	*SPHK1*	
	*SPHK2*	
S1P-phosphatase	*SGPP1*	
	*SGPP2*	
S1P lyase	*SGPL1*	
S1P-receptor	*S1PR1*	
	*S1PR2*	
	*S1PR3*	Association/trend with gene-related region^62,67^
	*S1PR4*	
	*S1PR5*	
Fatty acid desaturase	*FADS1*	
	*FADS2*	Association with SNPs in the gene^15,63,66^
	*FADS3*	Association/trend with gene-related region^64,107^
N-SMase activation associated factor	*NSMAF*	
Ceramide transfer protein	*COL4A3BP*	

#### Targeted analysis of common and rare variants in the case–control-based MTA cohort

We conducted a targeted exploration for association between ADHD and allelic variants in the 58 previously curated genes, using single- and multi-locus additive, dominant, and recessive linear mixed-effect models (LMEMs)^70^. We allowed up to 20 steps in the backward/forward optimization algorithm. We used persistent ADHD cases (after a 3-year follow-up)^36^ as the affected phenotype in all models. LMEMs allow the inclusion of both fixed (genotype markers, sex, and age) and random effects (family or population structure), with the latter accounting for potential inbreeding by including a kinship matrix (which, in our case, was estimated between all pairs of individuals using markers excluded from the final analysis after linkage disequilibrium (LD) pruning). A single-locus LMEM assumes that all loci have a small effect on the trait, while a multi-locus LMEM assumes that several loci have a large effect on the trait^70^. These models are implemented in SVS 8.3.0. The optimal model was selected using a comprehensive exploration of multiple criteria, including the extended Bayes information criteria, the modified Bayes information criteria, and the multiple posterior probability of association. *P* values were corrected for multiple testing using the false discovery rate (FDR) method.

#### Replication analysis in the Paisa and US cohorts

The genes included in the replication analysis were selected under a disjunctive-inclusive criterion based on the significant associations found in the discovery cohort. All the models applied to the discovery cohort were also used in this cohort. The analyses used family-based association tests (FBATs) under the “no linkage, no association” hypothesis, with age and gender as co-variates. In the case of the analysis for the Paisa cohort of families, an extreme, multivariate phenotype consisting of comorbid ADHD, CD, and ODD was used to define the “affected” phenotype. This combination of phenotypes offers a higher statistical power compared with permutation tests and with using separate tests for each outcome with adjustment for multiple testing^71–73^. Complex phenotypes are assessed by FBAT (as implemented in the PBAT module of SVS 8.4.0) allowing testing of a combination of phenotypes (power set of phenotypes; i.e., from independent traits—singletons such as ADHD alone—to complex combinations) to boost the FBAT power in predicting structures and substructures of new-composed phenotypes defined by the parents’ genotypes transmission to children. Thus far, following a sequential ascertainment strategy, we explore complex structures in the Paisa sample and evaluated our initial positive findings in additional samples^74,75^. A *P* value of 0.1 was set as the significance level for replication^76^. Thus, we expect that maximum 10% of genetic variants associated with ADHD are false positives^77,78^.

#### Meta-analysis

We performed a gene-based meta-analysis using the resulting *P* values of the discovery and replication cohorts for each gene. We used the FDR-corrected *P* values from the single-locus LMEMs for the MTA cohort, and the PBAT-based *P* values for the Paisa and US cohorts. Further, we explored the performance of the 58 sphingolipid gene set in the most recent Psychiatric Genomics Consortium (PGC) ADHD GWAS meta-analysis (20,000 cases and 35,000 controls), which includes 11 PGC samples and 23 iPSYCH genotyping batches^17^. This represents the largest ADHD data set available to date, with a total number of markers of 8,047,421 included in the GWAS meta-analysis. SNPs within the targeted genes found to be associated with ADHD in our study (*GALC*, *CERS6*, *SMPD1*, *SMPDL3B*, *CERS2*, *FADS3*, *ELOVL5*, and *CERK*) were extracted, for a total of 2012. For each gene, *P* values for these SNPs were jointly plotted with those reported in our study. *P* values for SNPs within each particular target gene were combined using the Stouffer’s method^79^.

Gene-based analysis was performed using VEGAS-2^80^ under default settings and the Knowledge-based mining system for Genome-wide Genetic (KGG) studies v4.0^81^ (http://grass.cgs.hku.hk/limx/kgg/index.html), as implemented in the gene-based association test using extended Simes (GATES), effective chi-squared (ECS), and univariate gene-based tests^82^. Gene set-based analysis was also performed in KGG 4.0 as implemented in the LDRT procedure^83^.

## Results

### Targeted analysis of common and rare variants in the MTA cohort

**Fig. 1:**
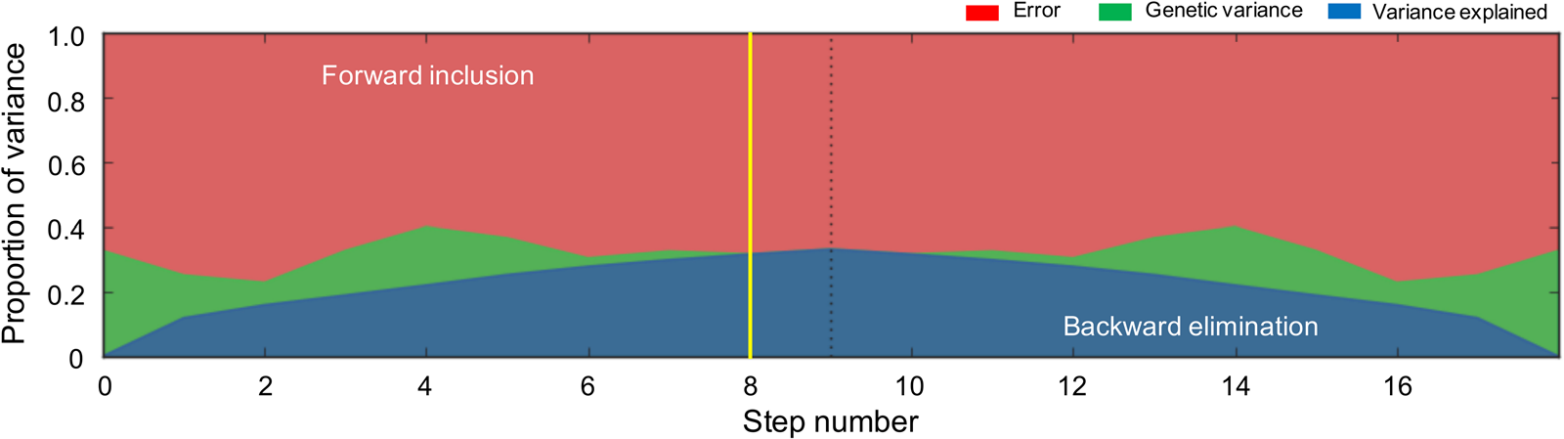
Partition of phenotypic variance in the single-locus linear mixed-effect models (LMEMs) for each forward inclusion (steps 1–9) and backward elimination (steps after the dotted line). The yellow vertical line marks the model selected based on the highest multiple posterior probability of association (mPPA) criterion.

Targeted screening in the MTA cohort included 137 informative markers, corresponding to rare and common allelic variants located in the 58 genes previously curated (Table 1). Single- and multi-locus additive, and dominant and recessive LMEMs were explored. Using the single-locus LMEM, we identified seven markers significantly associated with persistent ADHD (rs74073730 in *GALC*, rs4668077 in *CERS6*, rs35785620 in *SMPD1*, rs143078230 in *SMPDL3B*, rs139609178 in *CERS2*, rs200333847 in *FADS3*, and rs41273880 in *ELOVL5*) (Table 2a). Four of the eight genes associated with ADHD in the single-locus model were also associated in the multi-locus LMEM (*GALC*, *SMPD1*, *SMPDL3B*, and *CERS2*) (Table 2b). Additionally, an association for variant rs13057352 in *CERK* was found in the multi-locus model only (Table 2b). Optimization for the single-locus LMEM is presented in Fig. 1. The aforementioned model explained 30% of the phenotypic variance at step 8.

**Table 2:** Results of the association analysis for common/rare variants in MTA cohort by (A) single- and (B) multiple-locus linear mixed models.

(A)										
Chr	SNP	Position (hg19)	Gene	Marker information				Single-locus linear mixed model		
				Ref/Alt	MAF	CR	HGVS nomenclature	*β* (SE_*β*_)	*P* value	*P*_FDR_
14	rs74073730	88,429,817	*GALC*	G/A	0.016	1	c.1072C > T/p.Leu358Leu	0.52 (0.09)	1.65 × 10^−8^	2.26 × 10^−6^
2	rs4668077	169,439,848	*CERS6*	G/A	0.251	1	c.407 + 22016A > G/intronic variant	0.11 (0.02)	6.47 × 10^−6^	4.43 × 10^−2^
11	rs35785620	6,415,704	*SMPD1*	G/A	0.004	1	c.1763C > A/p.Thr588Lys	0.58 (0.17)	6.6 × 10^−4^	3.01 × 10^−2^
1	rs143078230	28,285,155	*SMPDL3B*	T/C	0.002	1	c.556T > C/p.Tyr186His	0.91 (0.29)	1.95 × 10^−3^	5.0 × 10^−2^
1	rs139609178	150,939,279	*CERS2*	G/A	0.002	1	c.801C > A/p.Val267Val	0.91 (0.29)	1.95 × 10^−3^	4.4 × 10^−2^
11	rs200333847	61,646,921	*FADS3*	C/T	0.002	1	c.385G > A/p.Asp129Asn	0.91 (0.29)	1.95 × 10^−3^	3.8 × 10^−2^
6	rs41273880	53,135,449	*ELOVL5*	T/C	0.002	1	c.779A > G/p.Tyr260Cys	0.91 (0.29)	1.95 × 10^−3^	3.3 × 10^−2^

(B)										
Chr	SNP	Position (hg19)	Gene	Marker information				Multi-locus linear mixed model		
				Ref/Alt	MAF	CR	HGVS nomenclature	*β* (SE_*β*_)	*P* value	*P*_FDR_
14	rs74073730	88,429,817	*GALC*	G/A	0.016	1	c.1072C > T/p.Leu358Leu	0.47 (0.09)	8.03 × 10^−8^	1.1 × 10^−5^
11	rs35785620	6,415,704	*SMPD1*	G/A	0.004	1	c.1763C > A/p.Thr588Lys	0.56 (0.15)	1.6 × 10^−4^	5.6 × 10^−3^
1	rs143078230	28,285,155	*SMPDL3B*	T/C	0.002	1	c.556T > C/p.Tyr186His	0.98 (0.25)	1.01 × 10^−4^	6.9 × 10^−3^
1	rs139609178	150,939,279	*CERS2*	G/A	0.002	1	c.801C > A/p.Val267Val	0.98 (0.25)	1.01 × 10^−4^	4.65 × 10^−3^
22	rs13057352	47,095,235	*CERK*	C/A	0.027	1	c.918G > T/p.Leu306Phe	0.21 (0.06)	1.51 × 10^−3^	3.45 × 10^−2^

*Chr* chromosome, *SNP* single-nucleotide polymorphism, *Ref/Alt* reference/alternate allele, *MAF* minor allele frequency in this cohort, *CR* call rate, *β* regression coefficient, *SE*_*β*_ standard error of *β*, *PP* value, *FDR* false discovery rate, *HGVS* Human Genome Variation Society.

### Sequential analysis in the Paisa and US cohorts

Eight genes (*GALC*, *CERS6*, *SMPD1*, *SMPDL3B*, *CERS2*, *FADS3*, *ELOVL5*, and *CERK*) were selected according to a disjunctive-inclusive criterion to be sequentially analyzed in the replication cohorts. Note that replication cohorts in this study correspond to independent samples differing in ethnic composition, recruitment strategy, and investigation timeframe. Thus, these cohorts are not intended for “exact replication,” but for a validation of the genetic associations in the discovery cohort under modified influencing factors (also called “local replication”), which includes the markers originally identified plus other markers in the same region that were not necessarily part of the original experiment (for instance, they may be monoallelic or in very low frequency in the discovery cohort). “Local replication” is considered to confer stronger evidence regarding the generalizability of genetic associations^84–87^.

The results of the analysis performed in the Paisa and US cohorts are shown in Table 3. Associations between ADHD and allelic variants in *GALC*, *SMPD1*, and *CERS6* were observed in both replication cohorts. The association with variant rs4668077 in *CERS6*, initially observed in the discovery cohort, showed “exact replication” in the US cohort. Additionally, an association with variant rs13393173 was observed in the Paisa and US cohorts (although it was not originally detected in the discovery cohort). For *GALC*, associations with variant rs398607 and rs1805078 were observed in the Paisa and US cohort, respectively. Variant rs1805078, associated with ADHD in the US replication cohort, present evidence of LD (*D*′ = 1 in African, European, and Admixed American populations, 1000 genomes) with variant rs7407370, associated with ADHD in the discovery cohort, and with variant rs398607, associated with ADHD in the Paisa replication cohort (*D*′ = 1 in African, *D*′ = 0.97 in European, and *D*′ = 0.93 in Admixed American population, 1000 genomes). Note that Paisa population is geographically isolated and were genetically originated from the admixture of Caucasian men Amerindian women. Variant rs7407370, originally associated with ADHD in the discovery cohort, is monoallelic in European population (1000 genomes) and present MAF = 0.001 in Admixed American population. For *SMPD1*, variant rs7951904 was found to be associated with ADHD in both replication cohorts. This variant present evidence for LD with variant rs35785620, originally associated with ADHD in the discovery cohort (*D*′ = 1 in African and Admixed American populations). Variant rs35785620, originally associated with ADHD in the discovery cohort, is monoallelic in European population (1000 genomes) and present MAF = 0.006 in admixed American population.

**Table 3:** Results for replication in the Paisa and US cohorts using FBATs.

Cohort	Chr	SNP	Position	Gene	Allele	Freq^a^	HGVS Cod/Prot	*P*_FBAT_
Paisa	14	rs398607	88,407,888	*GALC*	G	0.38	c.1685T > C/p.Ile562Thr	4.0 × 10^−2^
	11	rs7951904	6,412,931	*SMPD1*	G	0.1	c.636T > C/p.Asp212Asp	7.2 × 10^−2^
	2	rs13393173	169,389,091	*CERS6*	A	0.16	c.171 − 15015G > A/intronic variant	9.9 × 10^−2^
US	2	rs4668077	169,439,848	*CERS6*	A	0.18	c.407 + 22016A > G/intronic variant	1.1 × 10^−2^
	14	rs1805078	88,450,770	*GALC*	A	0.058	c.550C > T/p.Arg184Cys	3.9 × 10^−2^
	2	rs13393173	169,389,091	*CERS6*	A	0.22	c.171 − 15015G > A/intronic variant	4.4 × 10^−2^
	11	rs7951904	6,412,931	*SMPD1*	G	0.13	c.636T > C/p.Asp212Asp	7.9 × 10^−2^

*Chr* chromosome, *SNP* single-nucleotide polymorphism, *CR* call rate, *P* FBAT-based *P* value, *FDR* false discovery rate, *HGVS* Human Genome Variation Society, *FBAT* family-based association test.

^a^As estimated in these cohorts.

Meta-analysis of *P* values for the *GALC*, *CERS6*, and *SMPD1* genes were obtained based on both the Stouffer’s^79^ and Fisher’s^88^*P* value combination methods. Thus, the combined *P* value for the *GALC*, *CERS6*, and *SMPD1* genes were 1.44 × 10^–6^, 1.15 × 10^–3^, and 8.1 × 10^–3^, respectively, which all are significant at 5% even after correcting for multiple testing using the FDR method. These results suggest that the association between variants within these genes and ADHD is not unique to the MTA cohort, but can also be expanded to the Paisa and US cohorts.

Following this lead, SNPs within the target genes (*GALC*, *CERS6*, *SMPD1*, *SMPDL3B*, *CERS2*, *FADS3*, *ELOVL5*, and *CERK*) that were found to be associated with ADHD in our study were extracted from the PGC data set, as described in the “Methods” section (a total of 2012 SNPs). Combined analysis of SNPs in each gene in the PCG data set and in our study using the Stouffer’s method (which does not correct for markers in LD detected a strong association between ADHD and markers in *CERS6* (*P* < 0.0001), *SMPD1* (*P* = 0.0130), and *SMPDL3B* (*P* = 0.0034) from the PGC data set (Supplementary Fig. S1 and Supplementary Table S1).

Gene-level analysis identified three SNPs associated with ADHD (*CERS6-*rs183574665, *P* = 0.005; *SMPDL3B*-rs11577165, *P* = 0.022; *CERK*-rs9616098, *P* = 0.010), but significance was lost after LD correction (Supplementary Table S2). Likewise, the GATES and ECS methods did not yield a significant association between targeted genes and ADHD (Supplementary Table S2). There was no significant association after correction using the FDR method^89,90^.

### The rs398607 marker is associated with increased *GALC* mRNA expression in the cerebellum

Expression quantitative trait loci analysis of brain tissue from 137 neuropathologically confirmed controls (age 16–102) revealed a significant association between the rs398607 GG risk genotype and increased GALC expression in the cerebellum (*P* = 2.9 × 10^−8^) (Supplementary Fig. S2).

## Discussion

Sphingolipids are crucial for myelination and neurite outgrowth and maturation^28–30^, but their potential role as pathogenic factors in ADHD remains unexplored. Here, we present the first evidence supporting an association between variants in sphingolipid metabolism genes and ADHD risk.

**Fig. 2:**
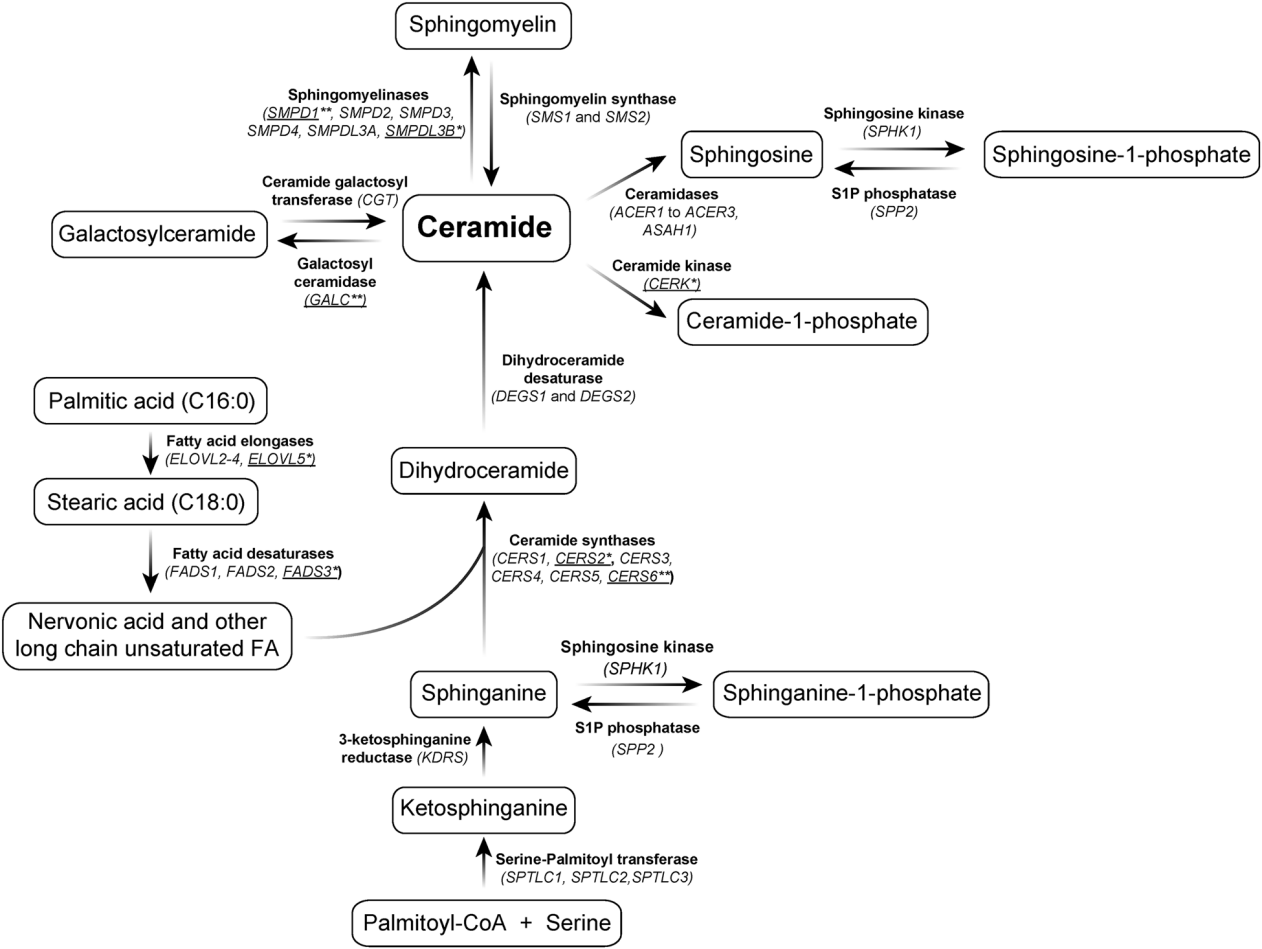
Schematic representation of sphingolipid and related fatty acid metabolism pathways. Genes from the sphingolipid pathway that were included in the targeted analysis are shown within parentheses. Genes significantly associated with ADHD are underlined (*significant association in the MTA cohort; **significant association in the MTA cohort and both replication cohorts). Additional reactions involving metabolism of glucosylceramide and related sphingolipids are not shown.

Figure 2 shows the main enzymes involved in sphingolipid biosynthesis and breakdown. Ceramide is central in sphingolipid metabolism and can be produced via either de novo synthesis or recycling pathways^91^. In de novo synthesis, ceramides are generated from serine and palmitoyl-CoA. In this pathway, ceramide synthases (CerSs) catalyze the acylation of sphinganine to produce dihydroceramides. Six types of CerS exist in mammals (*CERS1–6*), all of which are expressed in the brain, except for *CERS3*^92^. Because CerSs are length specific for fatty acyl-CoAs, they determine the length of downstream sphingolipids, including ceramides themselves, sphingomyelins and glycosphingolipids. Our targeted analysis on the genetic data from the MTA cohort found significant associations between ADHD (persistent phenotype) and variants rs4668077 (*P* = 6.47 × 10^−6^; *P*_FDR_ = 4.43 × 10^−4^; Table 2a) and rs139609178 (*P* = 1.01 × 10^−4^; *P*_FDR_ = 4.65 × 10^−3^; Table 2b) in the genes encoding for CERS6 and CERS2, respectively. The association between ADHD and variant rs4668077 in the *CERS6* gene was further replicated in the US cohort. Of note, CERS6-deficient mice present a hyperactive behavior^93^.

In the recycling salvage pathway, ceramides are generated from sphingomyelins and other complex sphingolipids (glycosphingolipids). Since sphingomyelins are the most abundant complex sphingolipids in human cell membranes, regulation of its metabolism is essential for cellular homeostasis. Breakdown of sphingomyelin occurs through the hydrolysis of phosphocholine head groups by enzymes from the sphingomyelinase family^94^. Our targeted analysis on the MTA genetic data detected association between ADHD and variants in the *SMPD1* and *SMPDL3B* (Table 2b) genes from the acid sphingomyelinase family. Allelic variants in *SMPD1* were also associated with ADHD in the Paisa and the US cohorts. Although the marker initially identified in the MTA cohort was not exactly replicated, the marker identified in replication cohorts is in close LD with variant rs35785620, originally associated with ADHD (*D*′ = 1 in African and Admixed American populations) (Supplementary Table S3.) *SMPD1* encodes for sphingomyelin phosphodiesterase 1 (acid sphingomyelinase), enzyme that has been implicated in the pathology of Niemann–Pick types A and B lysosomal storage disorders (MIM 257200 and 607616), inherited as autosomal recessive traits. Both disorders present with severe neurological involvement. In addition, in vitro and in vivo models have demonstrated a role of *SMPD1* in the pathogenesis of common complex neurologic disorders, such as depression and Alzheimer’s disease, highlighting the importance of acid sphingomyelinase in neurocognitive functioning in humans^95^. *SMPD1* marker rs35785620, significantly associated with ADHD in the MTA/discovery cohort, corresponds to a rare variant with a minor allele frequency (MAF) of 0.4%. This variant corresponds to a missense change leading to a threonine-to-methionine substitution at position 588 (NP_000534.3) and is predicted by Mutation Taster to disrupt the formation of a disulfide bond between cysteines at positions 586 and 590. The variant was predicted to have a neutral effect by PROVEAN. Variant rs143078230 in *SMPDL3B*, significantly associated with ADHD in the MTA cohort, corresponds to a rare missense variant (MAF = 0.2%), leading to a tyrosine-to-histidine substitution at position 186 (NP_001291508.1). The T186H change is predicted to be deleterious by both Mutation Assessor and PROVEAN.

Also, in the recycling pathway, targeted analysis of the MTA genetic data detected significant associations between ADHD and *GALC*, which codes for galactosylceramidase, enzyme responsible for the breakdown of galactosyl- and lactosylceramide, galactosylsphingosine, and galactocerebrosides. GALC defects lead to the accumulation of cytotoxic galactosylsphingosine (psychosine) in Krabbe disease^96^, an autosomal recessive disorder that results in demyelination and severe progressive motor neuron degeneration. Of note, two missense variants were detected in association with ADHD in the Paisa (rs398607) and US (rs1805078) Cohorts. Variant rs398607 leads to an isoleucine-to-threonine substitution at position 562; and variant rs1805078 leads to an arginine-to-cysteine substitution at position 184. The functional impact of rs398607 (MAF = 38% in the Paisa cohort) is predicted as moderately deleterious by Mutation Assessor and as deleterious by PROVEAN. The predicted functional impact of variant rs1805078 (MAF = 5.8% in the US cohort) is low according to Mutation Assessor and deleterious according to PROVEAN. The neuroanatomic and neurofunctional correlates of these variants are unknown.

The markers identified here are not represented in the most recent genome-wide significant ADHD study done by the PGC^17^, as they did not genotype for rare variants. In order to validate the replicability of our results, we performed a meta-analysis using SNPs from the PGC data set that were harbored in our ADHD-associated genes. To our satisfaction, we were able to detect moderate associations at the marker level in *CERS6*, *SMPD1*, and *SMPDL3B*, and at the gene level in *CERS6*, *SMPDL3B*, and *CERK*. Although genome-wide significance was not achieved, this result supports the findings of our study. It is important to mention, however, that while genome-wide association studies are a useful tool for discovering novel risk variants (as it involves a hypothesis-free interrogation of the entire genome) any lack of genetic association may just reflect the polygenic, multifactorial nature of ADHD, with both common and rare variants likely contributing to small genetic effects^97–99^. In addition, an important factor is the genetic heterogeneity of ADHD subtypes, which may have different underlying genetic mechanisms. Therefore, genome-wide significance may be achieved only for loci with larger genetic effects, while others with smaller effects remain undetected for a given population size.

Interestingly, the rs398607 risk allele was associated with increased *GALC* expression in the cerebellum. This makes sense functionally as more GALC activity would be intuitively associated with increased cerebellar myelin breakdown. Brain imaging studies have implicated cerebellar structural abnormalities in ADHD^100,101^. In addition to its role in motor control, the cerebellum contributes to a wide range of cognitive and affective processes. Lesion studies demonstrate important roles for the cerebellum in motor and perceptual tasks in which events span milliseconds—thus requiring fine temporal control^102^—in the orientation of spatial attention^103^, in verbal working memory and language processing^104^, and in affective regulation^105^.

Additional research on sphingolipid metabolism may shed light into the pathogenesis of ADHD in the context of detailed brain imaging evaluation of affected individuals. Although more research is needed, diffusion tensor imaging (DTI) has proven to be a promising technique for the diagnosis of white matter structural abnormalities in ADHD, consistent with fronto-striatal-cerebellar deficits^106^. The sensitivity of DTI to detect subtle changes in white matter integrity can provide a useful technique to investigate white matter tracts longitudinally in patients with ADHD in the context of sphingolipid genetic variation. Such research would provide new prospects and challenges for future research into the pathophysiology of ADHD.

## Conclusions

Sphingolipids are highly abundant in CNS and crucial to glial and neuronal function and development. To date, an association between ADHD and variation in sphingolipid metabolism genes had not been explored. Here we present the results from a targeted analysis of 58 genes directly involved in sphingolipid metabolism performed on three different cohorts from disparate geographical regions. We found an association between ADHD and variants in eight of these genes (*GALC*, *CERS6*, *SMPD1*, *SMPDL3B*, *CERS2*, *FADS3*, *ELOVL5*, and *CERK*) in the discovery cohort, with “local replication” for associations with variants in *CERS6*, *SMPD1*, and *GALC* genes. Some of these variants correspond to missense mutations with predicted damaging effects. This is the first piece of evidence linking genetic variation in sphingolipid metabolism genes to ADHD pathophysiology.

## Supplementary information

Supplementary Material
