# Supplementary material for: Mutations in sphingolipid metabolism genes are associated with ADHD
Source: Transl Psychiatry. 2020 Jul 13;10:231. doi: 10.1038/s41398-020-00881-8 (PMC7359313; doi:10.1038/s41398-020-00881-8)

Supplementary Material

Mutations in Sphingolipid Metabolism Genes Are Associated with ADHD

Marcela Henriquez-Henriquez, Maria T. Acosta, Ariel F. Martinez, Jorge I. Vélez, Francisco Lopera,

David Pineda, Juan D. Palacio, Teresa Quiroga, Tilla S. Worgall, Richard J. Deckelbaum,

Claudio Mastronardi, Brooke S. G. Molina and the MTA Cooperative Group,

Mauricio Arcos-Burgos, and Maximilian Muenke

Table S1. Result of SNP-based association analysis for targeted genes in the PGC data set.

Gene	Number of SNPs in PGC data set	-log10(*P*-value)		SNP-based combined *P*-value[a]
		Min (SNP)	Max (SNP)	
GALC	676	0.0013 (rs75360027)	2.6660 (rs8008772)	1.0000
CERS6	822	0.0011 (rs10490712)	2.3614 (rs10490712)	<0.0001
SMPD1	7	0.4306 (rs1050228)	0.8539 (rs1050239)	0.0130
SMPDL3B	74	0.0168 (rs57607983)	1.6538 (rs11577165)	0.0034
CERS2	29	0.1058 (rs2280627)	0.9488 (rs525676)	0.9502
FADS3	37	0.0134 (rs76656467)	1.0835 (rs75306442)	0.9998
ELOVL5	255	0.0003 (rs70980834)	0.9270 (rs209512)	0.9376
CERK	112	0.0057 (rs135692)	2.6558 (rs74486714)	0.9786
Total	2012			

[a]Estimated using Stouffer's method (Stouffer et al, 1949).

Table S2. Results of the gene-based association test for target genes in the PGC data set.

Gene	VEGAS-2		KGG 4.0 *P*-value	
	**Gene-based *P*-value**	**Top SNP (*P*-value)**	**GATES**	**ECS**
GALC	0.973	rs117778022 (0.075)	0.9968	0.89619
CERS6	0.099	rs183574665 (**0.005**)	[a]	[a]
SMPD1	0.182	rs1050239 (0.140)	[a]	[a]
SMPDL3B	0.206	rs11577165 (**0.022**)	[a]	[a]
CERS2	0.956	rs11579289 (0.522)	[a]	[a]
FADS3	0.929	rs75306442 (0.083)	[a]	[a]
ELOVL5	0.606	rs209512 (0.118)	[a]	[a]
CERK	0.746	rs9616098 (**0.010**)	[a]	[a]

[a]Not reported. VEGAS-2: Versatile gene-based association study version 2; KGG: Knowledge-based mining system for Genome-wide Genetic studies; GATES: Gene-based association test using extended Simes procedure; ECS: Effective chi-squared test.

Table S3. Pairwise linkage disequilibrium coefficients from the 1000 Genomes dataset.

Chr	Gene		AFR[a]			AMR[b]			EUR[c]		
			D' (r^2)			D' (r^2)			D' (r^2)		
			**rs74073730**	**rs398607**	**rs1805078**	**rs7407730**	**rs398607**	**rs1805078**	**rs74073730**	**rs398607**	**rs1805078**
14	*GALC*	**rs74073730**	-	1 (0.1329)	1 (0.0001)	-	1 (0.0009)	1 (0.0001)	-	NA	NA
		**rs398607**	1 (0.0706)	-	1 (0.0008)	1 (0.0009)	-	0.93 (0.051)	NA	-	0.97 (0.065)
		**rs1805078**	1 (0.0001)	1 (0.0008)	-	1 (0.0001)	0.93 (0.051)	-	NA	0.97 (0.065)	-
11	*SMPD1*		**rs7951904**	**rs35785620**		**rs7951904**	**rs35785620**		**rs7951904**	**rs35785620**	
		**rs7951904**	-	1 (0.2302)		-	1 (0.0525)		-	NA	
		**rs35785620**	1 (0.2302)	-		1 (0.0525)	-		NA	-	
6	*CERS6*		**rs13393173**	**rs4668077**		**rs13393173**	**rs4668077**		**rs13393173**	**rs4668077**	
		**rs13393173**	-	0.52 (0.027)		-	0.2 (0.025)		-	0.39 (0.093)	
		**rs4668077**	0.52 (0.027)	-		0.2 (0.025)	-		0.39 (0.093)	-	

[a]Minor allele frequency (MAF) in AFR (1000 genomes) rs74073730= 0.068; rs398607= 0.355; rs1805078= 0.002; rs7951904= 0.212; rs35785620= 0.058; rs13393173= 0.014; rs4668077= 0.27

[b]MAF in AMR (1000 genomes) rs74073730= 0.001; rs398607= 0.379; rs1805078=0.035; rs7951904= 0.099; rs35785620= 0.006; rs13393173= 0.148; rs4668077= 0.277

[c]MAF in EUR (1000 genomes) rs74073730= 0; rs398607= 0.488; rs1805078=0.062; rs7951904= 0.124; rs35785620= 0; rs13393173= 0.24; rs4668077= 0.16

AFR =African; AMR = Admixed American; EUR = European

Figure S1. GWAS meta-analysis *P*-values for the PGC data set SNPs harbored sphingolipid metabolism genes found to be associated with ADHD in our study. FBAT-based raw *P*-values for SNPs associated with ADHD in the MTA, Paisa and US samples are shown in color. For more details, see Tables 2 and 3 of the main manuscript.

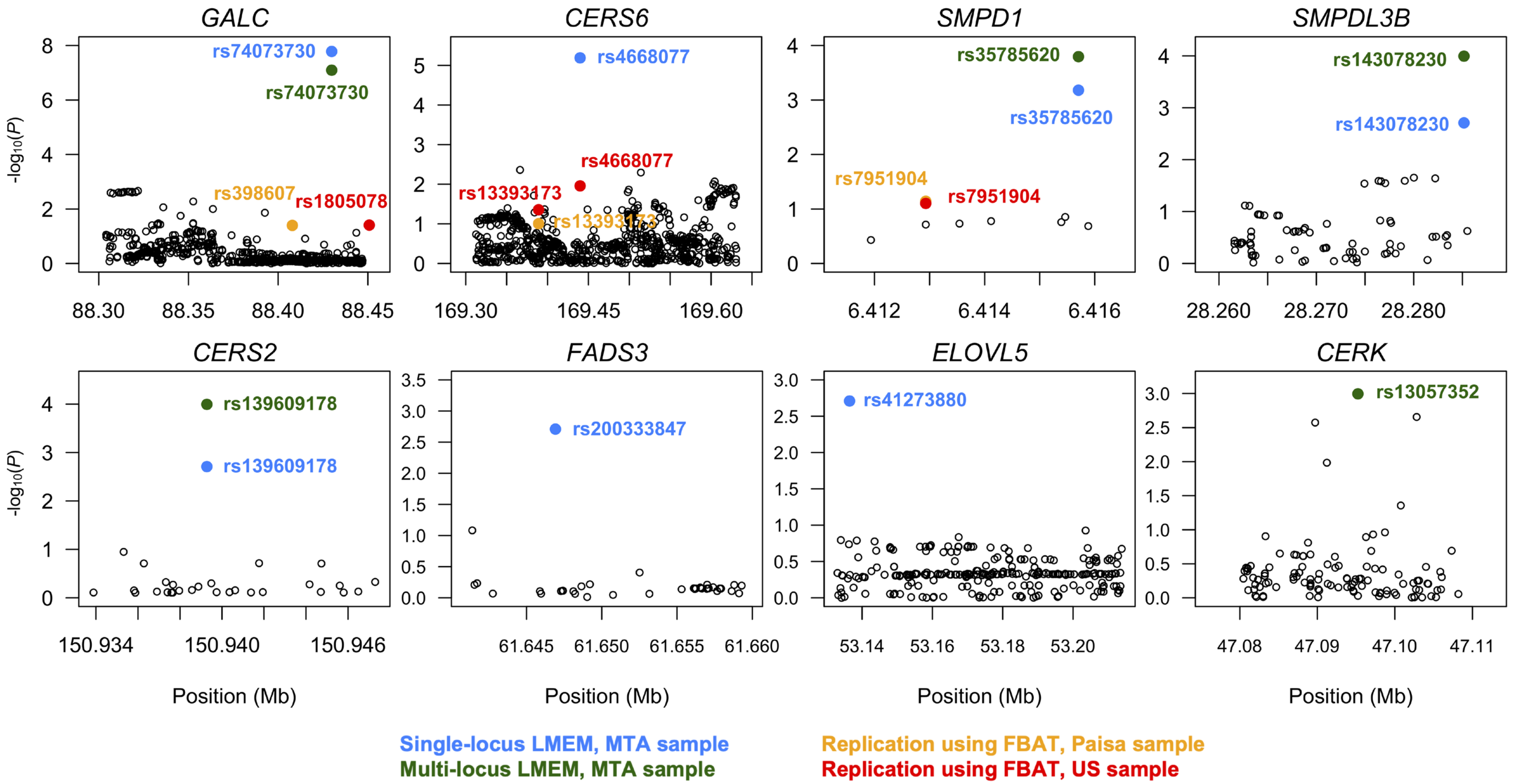

Figure S2. GALC mRNA expression in human post-mortem tissue is influenced by ADHD risk variant rs398607. Risk variant G is significantly associated with increased GALC expression in the cerebellum ($P=2.9 \times 10^{-8}$) (https://gtexportal.org/home/snp/rs398607).

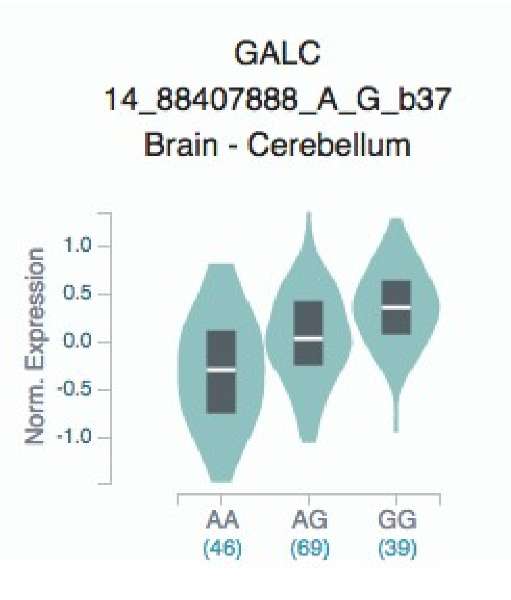

Supplement: Supplementary file 1 — Supplementary Material [file 41398_2020_881_MOESM1_ESM.pdf]
